# Targeting the Copper Transport System to Improve Treatment Efficacies of Platinum-Containing Drugs in Cancer Chemotherapy

**DOI:** 10.3390/ph14060549

**Published:** 2021-06-08

**Authors:** Macus Tien Kuo, Yu-Fang Huang, Cheng-Yang Chou, Helen H. W. Chen

**Affiliations:** 1 Department of Translational Molecular Pathology, The University of Texas MD Anderson Cancer Center, Houston, TX 77030, USA; tienkuo@sbcglobal.net; 2 Department of Obstetrics and Gynecology, National Cheng Kung University Hospital, College of Medicine, National Cheng Kung University, Tainan 704, Taiwan; yufangh@mail.ncku.edu.tw; 3 Department of Radiation Oncology, National Cheng Kung University Hospital, College of Medicine, National Cheng Kung University, Tainan 704, Taiwan; 4 Center of Applied Nanomedicine, National Cheng Kung University, Tainan 701, Taiwan

**Keywords:** cisplatin, carboplatin, oxaliplatin, copper transporter, Ctr1, Atox1, ATP7A. ATP7B. Sp1, copper chelator

## Abstract

The platinum (Pt)-containing antitumor drugs including cisplatin (cis-diamminedichloroplatinum II, cDDP), carboplatin, and oxaliplatin, have been the mainstay of cancer chemotherapy. These drugs are effective in treating many human malignancies. The major cell-killing target of Pt drugs is DNA. Recent findings underscored the important roles of Pt drug transport system in cancer therapy. While many mechanisms have been proposed for Pt-drug transport, the high-affinity copper transporter (hCtr1), Cu chaperone (Atox1), and Cu exporters (ATP7A and ATP7B) are also involved in cDDP transport, highlighting Cu homeostasis regulation in Pt-based cancer therapy. It was demonstrated that by reducing cellular Cu bioavailable levels by Cu chelators, hCtr1 is transcriptionally upregulated by transcription factor Sp1, which binds the promoters of Sp1 and hCtr1. In contrast, elevated Cu poisons Sp1, resulting in suppression of hCtr1 and Sp1, constituting the Cu-Sp1-hCtr1 mutually regulatory loop. Clinical investigations using copper chelator (trientine) in carboplatin treatment have been conducted for overcoming Pt drug resistance due in part to defective transport. While results are encouraging, future development may include targeting multiple steps in Cu transport system for improving the efficacies of Pt-based cancer chemotherapy. The focus of this review is to delineate the mechanistic interrelationships between Cu homeostasis regulation and antitumor efficacy of Pt drugs.

## 1. Introduction

Platinum (Pt)-based antitumor agents, including cisplatin (cis-diamminedichloroplatinum II, cDDP), carboplatin, and oxaliplatin, are active against many tumor types. cDDP is commonly used for treating metastatic testicular cancer, carboplatin for advanced ovarian cancer, and oxaliplatin for advanced colorectal cancer. cDDP has also been used for treating other cancers, including cancers of lung, bladder, head, and neck, and uterine cervix [1,2]. The use of cDDP is limited by its adverse side effects including nephrotoxicity [3,4], ototoxicity [4], and peripheral neurotoxicity. These toxicities are not associated with carboplatin, which mainly induces myelosuppression; whereas the most common toxicity associated with oxaliplatin is peripheral neuropathy [5].

The cytotoxic target of these drugs is DNA, by forming primarily intra-stranded crosslinks d(GpG) adducts. If not repaired, these lesions will damage DNA replication, transcription, and associated cellular functions, resulting in cell death or apoptosis. Previous work demonstrated that the overall lethality of drugs is positively correlated with the cellular contents of Pt drugs [6]. Moreover, it has been reported that reduced Pt levels are the hallmark of Pt-drug resistance [7]. These findings strongly suggest that Pt drug transport mechanisms play important roles in cell-killing of Pt-based antitumor agents. This review aims to address Pt drug transport mechanisms, focusing specifically on the roles of copper (Cu) transport system. We will discuss recent works relevant to improving Pt drug cancer chemotherapy by targeting the Cu transport system in cultured cell models and in clinical settings.

## 2. The Transport Mechanisms of Pt Drugs

Several mechanisms have been proposed for Pt drug delivery into cancer cells. Early studies suggested that cDDP enters cells by simple diffusion and independent of membrane protein carrier [8]. Later, using genome-wide loss-of-function screening of a haploid cell line, it was identified that loss of subunits leucine-rich repeat-containing protein (LRRC8A) and LRRC8D of the heteromeric LRRC8 volume-regulated anion channels (VRACs) increased resistance to cDDP and carboplatin but not oxaliplatin [9], perhaps due to structural differences among these drugs. Analyses of The Cancer Genome Atlas (TCGA) database showed that low LRRC8D expression correlates with reduced survival of Pt drug-treated ovarian cancer patients. VRACs are important regulators that control cellular volume-associated chloride and organic osmolytes movements across the cell membrane [10]. Moreover, incorporation of LRRC8D subunit into VRAC enhanced permeability for cDDP. Furthermore, it was reported that cDDP accumulation correlates with LRRC8A protein level and channel activity [11]. However, a recent study reported that knockdown or overexpression of LRRC8A and LRRC8D in a lung adenocarcinoma cell model did not affect cDDP resistance or sensitivity [12]. Elucidation of detailed mechanistic basis underlying how VRAC-mediated cDDP permeation into cells may resolve the discrepancies.

Multiple transporters have been reported for oxaliplatin, i.e., the organic cation transporter system (SLC22A), including OCT2 (*SLC22A2*) [13] and OCT3 (*SLC22A3*) [14], organic cation/carnitine transporters OCTNs (OCTN1/OCTN2) [15], and multidrug resistance protein 4 (MRP4) [16]. OCTs are a group of poly-specific transporters for subtracts typically of positively charged or zwitterions at physiological pH conditions, e.g., organic amines choline, neurotransmitters dopamine and serotonin, and vitamin B (thiamine) [17]. In one study, it was reported that long progression-free survival (PFS) in metastatic colon cancer treated with oxaliplatin is linked to high expression of OCT2 [18], implicating the role of this transporter in oxaliplatin treatment.

## 3. Connecting the Essential Trace Element Cu to Pt-Based Antitumor Drugs

### 3.1. Identification of the High-Affinity Copper Transporter (Ctr1) as cDDP Importer

Ishida et al. [19] using mutagenesis screening identified that the high-affinity copper transporter (Ctr1) is a cDDP uptake transporter in the yeast. These authors also demonstrated that disruption of y*Ctr1* showed reduced accumulation of cDDP and increased cDDP resistance. Similar results were found in murine cell line with deletion of the *mCtr1* alleles [19].

Ctr1 is an evolutionarily conserved Cu(I) ion transporter from yeast to humans. The h*Ctr1 (SLC31A1)* encodes a polypeptide of 190 amino acids, organized in three transmembrane domains with the N-terminus extracellularly located. Natural Cu exists in oxidized form i.e., Cu(II). It has to be reduced by reductases to Cu(I) for hCtr1 transport and the subsequent intracellular distributions and final export. Unlike yeast Ctr1, which uses iron-copper reductase Fre1p/2p [20], human membrane-associated reductase is not known. However, using model peptides, it was suggested that hCtr1 ATCUN (Amino Terminal Cu(II)- and Ni(II))-binding site located at the extracellular N-terminus and its adjacent bis-His sequences capture extracellular Cu(II) and converts it into Cu(I) in the presence of ascorbate [21]. However, whether ATCUN motif is involved in cellular hCtr1-mediated Cu(I) transport is unknown.

Biochemical analyses identified three highly conserved methionine (Met or M)-rich motifs (^7^MxMxxM, ^40^MMMMxM, and ^150^Mxxx154M) in hCtr1. The first two Met-rich motifs are located at the extracellular N-terminus, whereas the last Met-rich motif is located at the end of the second transmembrane domain (TM2) [22]. Site-directed mutagenesis revealed that these amino acids are also important for cDDP transport [23]. Two dimensional electron crystallographic data [24] revealed that a functional hCtr1 consists of three monomers in an ion channel-like structure with an ~8 Å distance in the central pore. Recently, X-ray crystal structure determination of a genetically engineered fish (*Salom salar*) sCtr1 revealed two residues in TM2 (M146 and M150), which are critical for Cu(I) binding through Cu-S coordination. These two Met-residuals are also critical for the metal specificity, i.e., Cu(I) vs. Cu(II), Na^+^, and K^+^, and for ion (substrate) passing [25].

The molecular mechanisms underlying cDDP transport by hCtr1 are largely unknown. At face value, the molecular size of cDDP (chemical formula: cis[Pt(Cl_2_(NH_3_)_2_) is larger (at least three to four orders of magnitude) than the size of the hCtr1 pore. It has been suggested that cDDP interacts with accessible methionines in the (Met)-rich motifs by forming [Pt(Met)Cl(NH_3_)_2_] intermediate, resulting in induction of hCtr1 conformational changes [23]. cDDP and Cu(I) are thought to transverse through the axis of the trimeric hCtr1 channel and move inward by an intermolecular sulfur-sulfur processive exchange, like the mechanism underlying Cu(I) and Ag(I) transports by the bacterial periplasmic CusA efflux pump [26]. Alternatively, a model of hCtr1-mediated endocytosis was proposed [27].

It is important to note that hCtr1 transports cDDP and carboplatin (with reduced rate) but not oxaliplatin and Pt(IV) antitumor drugs (Ormaplatin, iproplatin, etc.), likely due to apparent differences in molecular masses. The physico-chemical properties of Pt(IV) agents differ greatly from those of the Pt(II) compounds [28]. In clinical trials, Pt(IV) drugs are normally delivered by nanoparticles. Once inside the cells, Pt(IV) compounds are released and converted into classical Pt(II) forms. Thus, the Pt(IV) may be considered as Pt (II) prodrugs.

### 3.2. Cu Chaperones in Intracellular cDDP Trafficking

**Figure 1:**
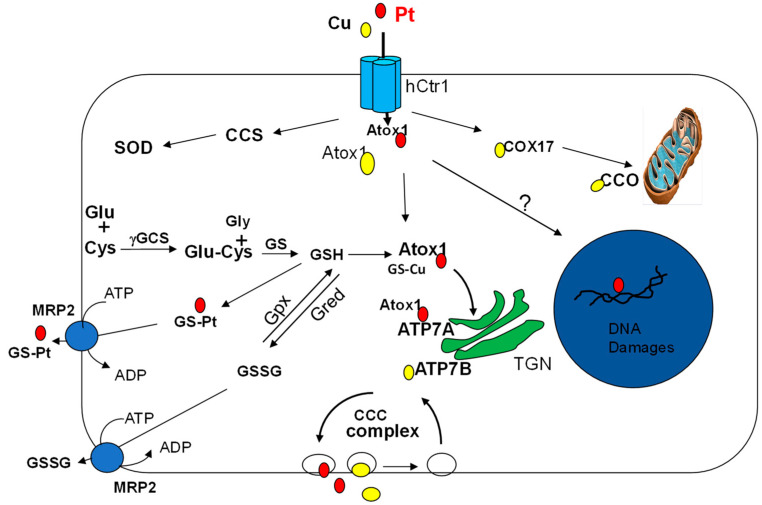
Schematic illustration of copper and platinum entrance, distribution, and elimination in tumor cell. Copper (Cu) and cDDP (Pt) entering cell via Ctr1 are transferred to Chaperones Atox1 and then to ATP7A/ATP7B at the Trans-Golgi Network (TGN) in the cytosol via the help of glutathione (GSH). GSH is synthesized via two sequential enzymatic reactions: ligation of glutamine (Glu) and cysteine (Cys) by γ-glutamylcysteine synthetase (γ-GCS), followed by the addition of glycine (Gly) by glutathione synthetase (GS). Interaction of ATP7A with CCC complex, which consists of COMMD/CCDC22/CCDC93 nodule to eliminate Cu or Pt out of the cell. Cu(I) and Pt can be transferred to mitochondrial cytochrome oxidase (CCO) or to Cu-Zn-superoxidase dismutase (SOD) via chaperones Cox17 and CCS, respectively. GSH and cDDP can form Pt(GS)2, which is eliminated by multidrug protein (MRP2) efflux pump. GSH is oxidized to GSSG by GSH peroxidase (Gpx) and GSSG is reduced back to GSH by GSH reductase (Gred). GSSG can be eliminated by MRP2 using ATP hydrolysis as an energy source.

Intracellular cDDP trafficking to different subcellular compartments generally follows the paths of Cu(I) distributions carried out by Cu chaperons, i.e., by cytochrome C oxidase copper chaperone Cox17 to mitochondrion, by superoxide dismutase 1 (SOD1) carrier (CCS) to cytoplasmic SOD1, and by antioxidant protein 1 (Atox1) to Cu-efflux pumps ATP7A and ATP7B, the two P-type ATPases located at trans-Golgi network (TGN) (Figure 1). Transcriptome analysis revealed that many of these Cu transporter proteins are coordinately regulated in cancer cells [29]. Atox1 is particularly relevant because it shuffles cDDP to the ATP7A/ATP7B efflux pumps. Atox1 is a soluble protein of 68 amino acids, which captures Cu(I) by directly interacting with the C-terminal ^188^HCH end of hCtr1. Atox1 coordinates one Cu(I) ion with the cysteine (Cys15) residues of the conserved ^12^CXX^15^C motif in Atox1 dimerization [30,31,32].

cDDP can also bind Cox17 at (Cys26 and Cys27) [33]. Moreover, in a fruit fly model, it was found that cDDP can bind CCS [34], also to the conserved ^12^CXX^15^C-Cu(I) binding motif [35], supporting that cDDP and Cu(I) share similar mechanisms in intracellular trafficking (Figure 1).

In addition, Atox1 contains a nuclear targeting signal (^38^KKTGK) for Cu-dependent nuclear translocation [36]. It has been shown that Atox1 may participate in the regulation of genes encoding superoxide dismutase 3 [37], pluripotency factor OCT4 [38], and NADPH oxidase organizer p47phox [39]. Deletion of Atox1 results in increased resistance to cDDP [40,41], perhaps due, at least in part, to impairment of nuclear targeting of Pt drugs to elicit DNA damages of the lethal effects [42].

### 3.3. Cu-ATPases in Pt Drugs Efflux

The two P-type ATPases ATP7A and ATP7B are well-known cellular Cu(I) exporters. ATP7A is mainly expressed in the intestinal epithelium for Cu absorption from food. ATP7A deletion results in systemic Cu deficiency that causes the Menkes’ disease. ATP7B is mainly located in the livers. Mutations of ATP7B result in massive Cu accumulation in the livers, resulting in Wilson’s disease [43]. Cell culture model revealed that ATP7A deletion enhances ATP7B expression, suggesting a compensatory expression between ATP7A and ATP7B [44].

The human ATP7A and ATP7B contain 1500 and 1465 amino acids, respectively, each organizing in eight transmembrane domains. The N-termini of ATP7A and ATP7B contain six metal-binding domains (MBD), each contains a CXXC motif that receives Cu(I) or cDDP delivered by Atox1. The folding of these CXXC MBD shares similarity with that in Atox1 that is believed to facilitate rapid intermolecular metal transfer between Atox1 and ATPases [45]. Moreover, the conserved amino acid ^60^lysine in Atox1 is critical for hetero-protein interactions between Atox1 and ATPases for processing Cu(I) transfer [45].

Several structural domains in ATP7A/ATP7B are important for the Cu(I) transport functions, i.e., an auto-activation domain (domain A) in the second cytosolic loop, a nucleotide-binding domain (N), and a phosphorylation domain (P) in the third cytosolic loop [46]. The A-domain is for actuator/dephosphorylation, the N domain for the ATPase catalytic activity, and the P domain for phosphorylation. While Cu-Atox1 potentially can interact with all six MBDs of ATP7A and ATP7B, it preferentially interacts with the MBDs 1 to 4 [47]. The binding induces conformational changes, activates ATPase catalytic activity by mobilizing MBDs to cross-talk with the N-domain of ATP7B [48], and induces autophosphorylation at ^1027^Asp in the P domain by ATP, and phosphorylation of several serine residues in the TMDs by protein kinase D (PKD). These result in the release of Cu(I) into the lumen of TGN. However, molecular mechanistic details of how Cu(I) travels from the cytosolic face and translocates through the membrane and is then released into the secretory compartment are largely unknown. A study using a Cu(I)-ATP ortholog (LpCopA) from *Legionella pneumophila* revealed a sulfur-lined metal transport pathway, involving a trigonal-planar coordination of Cu(I) with the Cys residues of the conserved CPC motif in transmembrane segment 4 (C382 and C384) and the conserved Met-residue of transmembrane segment 6 (M717 of the MXXXS motif) for Cu(I) exit pathway [49].

Under low Cu conditions, ATP7A and ATP7B are mainly associated with TGN [44]; whereas under elevated Cu conditions, they move via endosomal vesicle to peripheral membrane where Cu(I) is exported out of the cells. Both ATP7A and ATP7B actively recycle between the endosomal network and the plasma membrane by a molecular machinery called the CCD complex (COMMD/CCDC22/CCDC93) [50]. This complex consists of COMMD1 (the copper metabolism MURR1 domain 1) and coil-coil proteins CCDC22 and CCDC93. This complex is required for trafficking ATP7A from TGN to peripheral vesicles and then to the plasma membrane in high-Cu conditions [51] (Figure 1). COMMD1 is also required for ATP7B trafficking [52]. Hepatic deletions of COMMD1 results in accumulation of Cu and development of Cu toxicosis in the livers [50,53]. In contrast, intestinal deletion of COMMD1 (and COMMD9) did not show altered Cu levels in enterocytes. Moreover, these animals did not show reduced serum ceruloplasmin levels when fed ammonia tetrathiomolybdate, a specific Cu chelator that induces Cu deficiency in animals, demonstrating organ-specificity of COMMD’s functions [50].

It is difficult to study the molecular details of Cu(I) handling by the Cu-ATPases in intact living cells because of low expression levels of these ATPases and because of their complex structural configurations. A label-free bio-electrochemical in vitro method using microsomal vesicles containing recombinant ATP7B or ATP7A proteins immobilized onto a solid support membrane (SSM), which consists of layers of phospholipid, alkanethiol, and gold film, was developed. The SSM was developed to measure Cu ions movements. Using this approach, it was demonstrated that cDDP and oxaliplatin activate the ATPase cycle and, in the presence of ATP, Pt drugs translocate across the vesicles [54]. This methodology, while it did not directly measure Pt drug transport in living cells because of the limitations mentioned, it provided valuable information that otherwise would not be available. These results implicate that the gross mechanisms of Pt(II) transport by ATP7A and ATP7B are similar to the Cu(I) transport [55].

### 3.4. Redox Regulation of the Cu Transport System in Pt Drug Pharmacology

Copper is a redox-sensitive element, therefore, its physiology is greatly affected by cellular redox conditions. Copper is also an essential element for cell survival and growth, but excess Cu (normally exceeding 10 µM [56]) is detrimental. Therefore, cellular Cu levels must be exquisitely regulated. Cu catalyzes the Fenton reactions, which involve iron-mediated conversion of H_2_O_2_ to hydroxyl radicals [57]; both are important reactive oxygen species (ROS). ROS induce oxidative damage of nucleic acids, proteins and lipids. Excessive ROS is lethal, therefore, Cu homeostasis has to be constantly under the control of redox conditions.

One of the most abundant physiological antioxidants that regulates intracellular Cu redox conditions is glutathione (GSH). GSH is a tripeptide synthesized by sequential reactions involving glutamine (Glu) and cysteine (Cys) conjugation catalyzed by γ-glutamylcysteine synthesis (γGCS) followed by the addition of glycine (Gly) by glutathione synthetase (GS) (Figure 1). GSH is oxidized to GSSG by glutathione peroxidase (Gpx), and GSSG is reduced back to GSH by glutathione reductase (Gred). Ratios of GSH and GSSG mostly reflect the cellular redox conditions, which are variable in different cellular compartments.

Cellular GS and Gpx activities are critical in regulating GSH/GSSG ratios. Human GS is a homodimeric enzyme encoded by a single gene, whereas Gpx consists of a family of at least seven isozymes encoded by genes located on different chromosomes. Different isoforms have different tissue expression patterns [58].

Several mechanisms are involved in the regulation of Cu homeostasis by GSH: One, GSH can bind Cu(I) results in reduction of bioavailability of Cu(I) that leads to increased hCtr1 expression (see below) [59]. Binding of GSH by Cu(I) induces GSH depletion leading to increased ROS that stimulate expression of Gpx, which drives conversion of GSH to GSSG. GSSG is a substrate of multidrug protein (MRP2) efflux pump (Figure 1). Two, Cu(I) shuffles Cox17 to CCO in the mitochondrion, which is the powerhouse of ROS production. Third, GSH can assist conjugations between Cu(I) and Atox1 and facilitate their translocations to Cu-ATPases [60]. Interactions of cDDP with CCS and Atox1 are also redox-sensitive. GSH significantly modulates the platination of Cox17 [33,61]. Under oxidized conditions, the cysteine residues in the ^12^CXX^15^C of Atox1 form a disulfide bond that prevents Cu(I) from interaction.

Pt drugs are well-known for inducing ROS-related oxidative stress that enhances the expression of γGCS and GS, leading to increased biosynthesis of antioxidant GSH [62] (Figure 1). For many years, it was interpretated that the increased GSH was a determining mechanism that causes cDDP resistance [63]. However, we performed a transfection experiment using recombinant γGCS, the rate-limiting enzyme for the biosynthesis of GSH.

We found that increased GSH per se failed to confer cDDP resistance, but instead sensitizes the transfected cells to cDDP [64]. This is because GSH is a strong Cu(I) chelator that induces hCtr1 expression due to Cu homeostasis regulation (see [59] and below). These results further support the roles of redox-modulating system in Pt drugs cellular pharmacology.

Another aspect of redox regulation in Cu physiology is the recent finding that histone H3-H4 tetramer is an oxidoreductase that catalyzes the conversion of Cu(II) to Cu(I) in the nucleus in yeast *S. cerevisiae*, implicating chromosomal proteins in the maintenance of nuclear Cu(I) status. Intriguingly, the generated Cu(I) is functional in supporting Cu-utilizations in the cytosol, including mitochondrial respiration and cytosolic SOD activity [65]. Given the highly evolutionally conserved nature of histone proteins, they may play similar roles in mammalian cells. This may be of particular relevance in viewing the redox regulation of Pt-induced DNA damage and repair in the nucleus [66], which we know very little about it.

## 4. Modulations of Copper Homeostasis and Pt Drug Cancer Chemotherapy

### 4.1. Roles of the Copper Transport System in Pt Drug Cancer Chemotherapy in Clinical Settings

#### 4.1.1. hCtr1

Ishida et al. [67] analyzed an array-based hCtr1 expression dataset consisting of 91 patients with stage III or IV serous epithelial ovarian cancer who had been treated with a cytoreductive surgery followed by adjuvant chemotherapy of a Pt drug and a taxane deposited in TCGA. It was observed that elevated expression of hCtr1 was associated with increased disease-free survival. Similar results were obtained by analyzing an independent data set consisting of 285 patients [23]. Furthermore, in a meta-analysis of 12 studies with eight datasets in TCGA, it revealed that high hCtr1expression was significantly associated with a favorable overall survival (OS), progression-free survival (PFS), disease-free survival (DFS), and treatment response [68]. These results, collectively, strongly suggest that hCtr1 expression levels are correlated with cDDP efficacies in clinical settings.

cDDP has achieved a remarkable cure rate in advanced testicular cancer treatment [69]. The clinical mechanisms were investigated in animal models. Testis actively produces sperms in male fertilization. Spermatogenesis takes place in seminiferous epithelium, which consists of germ cells (GC) and Sertoli cells (SC). The somatic SC plays supportive roles in protection, nutrition, and proliferation of GC. Murine mCtr1 is highly expressed in SC [70], suggesting that mCtr1 in SC may contribute to the cDDP-induced testicular cell-killing. Indeed, SC-specific knock out of m*Ctr1* resulted in reduced sensitivity of testicular germ cells to cDDP treatment [71].

#### 4.1.2. Atox1, ATP7A and ATP7B

It has been reported that deletion of Atox1 resulted in inability of cDDP delivery to ATP7A/ATP7B [40,41]. This may be associated with increased cDDP sensitivity. As for the roles of ATP7A and ATP7B, many studies have demonstrated that elevated expression of ATP7A and ATP7B is associated with poor outcomes of Pt-based cancer chemotherapy in cancers of ovary, breast, and lung (see review in [72] and references therein). In other studies, overexpression of ATP7A and ATP7B was associated with resistance to cDDP [73,74,75], carboplatin [76] or oxliplatin [77]. Likewise, elevated ATP7B levels were correlated with worse treatment outcomes of cDDP in various cancers [75,78].

#### 4.1.3. COMMD1

COMMD1 is a multifunctional protein that interacts with many other proteins [79]. While the majority of COMMD1 is cytosolic, a small fraction is present in the nucleus. It was reported that elevated levels of nuclear COMMD1 confer sensitivity of ovarian cancer cells to Pt-based chemotherapy. Consistent with this in vitro finding, it was reported that increased expression of COMMD1 in the nucleus of ovarian tumors is associated with improved response to cDDP therapy [80].

### 4.2. Enhanced cDDP Cell-Killing Activity through Upregulation of hCtr1 Expression

The above observations indicate that almost all, if not the entire, processes of the copper transport system are involved in Pt-drug chemosensitivity regulation. These observations also suggest that targeting the Cu transport system may be an effective approach to improve Pt drug cancer therapy. In this review, we will focus on targeting hCtr1 expression.

It is important to note that the majority of cellular Cu is bound by cellular constituents and only a small fraction is free Cu for exchange. For example, about 90% of Cu in the circulation is bound by ceruloplasmin and the other 10% is bound by albumin, histidine, GSH, and transferrin [81,82]. At the intracellular level, in the entire process of Cu(I) transport system, from hCtr1 to the ATPases, Cu(I) movements involve directly coordinate transfers between donor and recipient molecules without metal dissociation. There is only a very small fraction that is loosely bound basal Cu pool or exchangeable Cu pool, which can be monitored using fluorescent sensor, Copper Fluor-3 imaging [83]. It is this labile Cu pool, but not the total cellular Cu content, that regulates hCtr1 expression. Regulation of Ctr1 expression by Cu bioavailability is an evolutionarily conserved mechanism from yeast [84] to humans [60].

Early work in the yeast *S. cerevisiae* demonstrated that Cu treatment induced yCtr1 internalization, which rapidly degraded [85]. Cu-induced hCtr1 internalization has also been reported in human cells [83,84].

#### 4.2.1. Transcriptional Regulation of hCtr1 Expression and cDDP Cancer Chemotherapy

Transcriptional regulation of yCtr1 has been well studied and involves two major transcription factors, Macp1 and Ace1. Ace1 responds to toxic Cu levels by transcriptionally upregulating detoxification genes *CUP1* and *CRS5,* which encode metallothionein; whereas Macp1 is the transcriptional regulator controlling the expression of yCtr1 and yCtr3 and reductase Fre1, together encoding the Cu import system in response to Cu-limiting conditions [86]. Both Macp1 and Ace1 contain Zinc-finger-like motifs and (K/R)GRP DNA binding motifs that interact with the promoters of their target genes. These transcriptional regulators also contain multiple Cys-rich segments in their C-termini that function as Cu(I)-sensing motifs [86,87].

Besides yeast, Cu homeostasis plays important roles in transcriptional regulation of host–pathogen interactions. The fungal pathogen *Cryptococcus neoformans* infected immunocompromised patients (such as HIV-infected individuals) by successful colonization of the lungs that can disseminate to the brain, causing lethal cryptococcal meningitis. The two Cu-detoxifying metallothionein proteins are activated in the lungs during infection; whereas Cu transporters Ctr1 and Ctr4 are critical for Cu acquisition and colonization in the brain. Transcription factor Cuf1 regulates the expression of these genes in response to Cu levels. Under low Cu concentrations, Cuf1 recognizes a Cu-responsive element (CuRE), which shares Macp1 recognition sequence; whereas at Cu elevated conditions, Cuf1 regulates Cu-detoxifying genes [88]. Cuf1 shares sequence similarities to the Ace1 and Macp1 including the N-terminal Zn-transcriptional binding motifs, highly conserved positively charged “KGRP” motif, and the Cys-rich Cu sensing segments. In this case, one transcription factor can sense both Cu-abundant and Cu-deficient conditions.

Transcriptional regulators of Ctr genes are also found in other organisms, including CRR1 factor for algae (*Chlamydomonas* sp.), which regulates 60+ genes including those encoding Cu transporters Cyc6, CPX1, CRD1, and redox proteins [89]; MTF-1 factor for the *Drosophila* dCtr1B transporter [24]; and SQUAMOS promoter-binding protein like 7 (SPL7) factor for the three Cu transporter genes COPT1, COPT2, and COPT6 in plant (*Arabidopsis* sp.) [90]. These transcriptional factors contain DNA-binding domains and unique Cu-binding domains that allow them to sense Cu ions [60,91].

#### 4.2.2. The Cu-Sp1-hCtr1 Inter-Regulatory Loop in Humans

**Figure 2:**
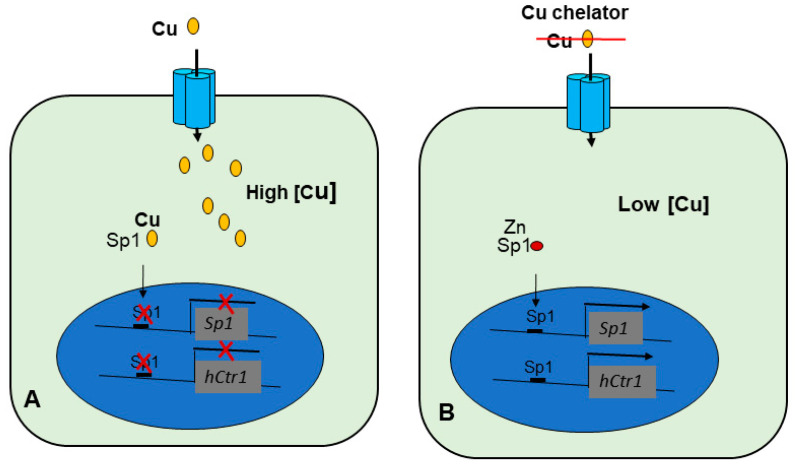
Models depicting regulation of Sp1 and hCtr1 expression by different Cu(I) levels. (**A**) Under high Cu(I) levels, Cu(I) displaces Zn(II) from the Zinc fingers of Sp1, resulting in inactivation of Sp1 and its inability for transcriptional regulation of *Sp1* and *hCtr1*. (**B**) In contrast, under low Cu conditions, Sp1 is capable of binding to the promoters of *Sp1* and h*Ctr1*, and transcriptionally induces their expression.

In humans, we previously reported that specificity protein 1 (Sp1) is the transcription factor that regulates hCtr1 expression in response to Cu conditions [92,93]. Unlike Cuf1, whose expression is not regulated by Cu concentration variations, Sp1 itself is transcriptionally regulated by Cu homeostasis. High Cu conditions downregulate Sp1 expression, whereas reduced Cu conditions upregulate Sp1. Sp1, in turn, regulates hCtr1 expression. Regulations of Sp1 and hCtr1 by Cu concentration variations are through Sp1-bindings at the promoters of these genes [93] (Figure 2).

Sp1 consists of a DNA-binding domain at the C-terminus that contains three zinc fingers (ZFs) and a transactivation domain that contains two serine/threonine-rich and two glutamine-rich (Q-rich 1 and Q-rich 2) subdomains. The ZF of Sp1 is constitutively bound by Zn(II) because apoSp1 is very unstable. Each ZF consists of Cys2-His2 residues that are coordinated by one Zn(II) molecule. Elevated Cu ions displace Zn(II) binding of Sp1 [94], rendering Sp1 unable for DNA binding. Cu can also disrupt nonclassical ZF-protein from RNA binding [95]. Thus, Cu is a negative regulator of Sp1 by poisoning ZF DNA binding domains. By the simple in vitro gel-electrophoretic DNA binding assay, it was demonstrated that elevated Cu(II) causes reduction of Sp1, whereas reduced Cu(II) levels by Cu-chelator, bathocuproine sulfonate (BCS), enhances Sp1-DNA binding. While this study was performed using Cu(II), it has been reported that Cu(I) can also displace Zn(II) from Sp1 [96]. These results demonstrated that a single transcription regulator Sp1 can control both up- and down-regulations of hCtr1 expression in Cu homeostatic response. Collectively, these results demonstrated that Cu(I) regulates Sp1, which transcriptionally regulates Sp1 and hCtr1, and hCtr1 feeds back to control cellular Cu levels, constituting the Cu(I)-Sp1-hCtr1 mutual regulation loop of Cu homeostasis [92].

Besides hCtr1, Sp1 is also known to regulate *ATP7A* through promoter binding [97]. Moreover, Sp1 also transcriptionally regulates *COMMD1* by binding to its promoter [98]. Although cDDP does not directly act upon Sp1, or very weakly at best, it was found that cDDP enhances Sp1-DNA binding in vitro [94], consistent with the previous observations that cDDP induces Sp1 and hCtr1 expression [99].

## 5. Overcoming cDDP Resistance by Copper Chelators

Many human cancers have increased Cu contents that are implicated in tumor angiogenesis, proliferation, and migration. Likewise, Sp1 expression is also frequently elevated in many human cancers [100]. Many preclinical studies have demonstrated that Cu chelators suppress tumor growth in cultured cell models [101] and in animal tumor models (see review [102] and references therein). Cu chelators used in these studies include tetrathiomolybdate (TM) [102], D-penicillamine (D-Pen) [102], trientine triethylenetetraminee dihydrochloride [102], disulfram (DSF) [103,104], and elesclomol (STA-4783) [105] (see reference [102] for the chemical structures and their clinical trials for some of these Cu chelators). TM, D-pen, and trientine are traditional medicines used in treating Wilson’s disease, whereas DSF is a conventional anti-alcoholism drug, and STA-4783 is an anti-neurodegenerative agent recently found to have activity of escorting Cu to the brain in mouse Menke’s disease model [106].

The Cu-Sp1-hCtr1 cycle underscores that the cellular levels of Cu(I), Sp1, and hCtr1 are mutually regulated. The capacities of cellular hCtr1 levels that can be regulated by Cu(I) depletion are constrained and may vary among cell types. For example, cells with reduced basal hCtr1 levels have higher magnitudes of hCtr1 upregulation by Cu chelators than those with elevated basal hCtr1 levels. Because most cDDP-resistant variants are associated with reduced hCtr1 expression [107], these results suggest higher magnitude of hCtr1 induction by chelators in cDDP-resistant cells than in their drug-sensitive counterparts. This predication was confirmed in our study using three cultured cell models and three different Cu-lowering agents (trientine, D-pen, and TM); cDDP-resistant cancer cells exhibit a greater magnitude of hCtr1 upregulation by the Cu-lowering agents as compared with their drug-sensitive counterparts. These observations indicate that reversal of cDDP resistance is independent of Cu-lowering agents and of cell line-specificities [108]. Another study using high-throughput screening identified that disulfiram exhibits synergistic effects with cDDP in bladder cancer cells [109]. Moreover, other naturally occurring Cu-lowering products include carnosine dipeptide (alanyl-L-histidine), which has been reported to modulate the Sp1-hCtr1-Cu homeostasis system [110], and curcumin (a product of plant *Curcuma longa* used in food flavoring), which enhances the binding of Sp1 to *Ctr1* and Sp1 promoters, and thus induces Ctr1 expression and chemosensitization to cDDP treatment [111]

Based on these preclinical observations, two clinical trials have been carried out in testing the efficacies of Cu-lowering agents as enhancers in Pt drug cancer chemotherapy: one (at MD Anderson Cancer Center) involved carboplatin plus trientine in 55 patients with advanced malignancies, 45 of which had prior failure in Pt drug treatment. The results showed that about 19% of patients (*n* = 9) who maintained low serum Cu levels after the treatments had significantly longer median PFS (*p* = 0.001) and OS (*p* = 0.03), as compared with those patients (*n* = 38) who did not [112,113]. The other study (at the National Cheng Kung University, Taiwan) involved carboplatin plus trientine and pegylated lyposomal doxorubicin in 18 Asian epithelial ovarian tubal and peritoneal cancers. The clinical benefit rate was 33.3 and 50.0% in the Pt-resistant and the partially Pt-sensitive group, respectively [114].

Savage therapy of Pt-resistant patients is well-known to produce low response rates (less than 10%) [115,116]. These studies provided first-in-human encouraging results that warrant further investigations.

## 6. Conclusions and Perspectives

Pt-based drugs represent an extraordinary accomplishment in inorganic antitumor drug development [2]. The discovery that Pt drugs transport is tightly regulated by the Cu transport system is intriguing, because the general physico-chemical properties between Pt(II) and Cu(I) do not have that much in common. Nonetheless, substantial understanding regarding how Cu importer hCtr1, Cu chaperone Atox1, and Cu exporters ATP7A/ATP7B handle Pt drugs in-and-out of the cells has been accumulated. However, much remains to be learned. While biochemical and genetical evidence has demonstrated that hCtr1 is involved in cDDP uptake, the precise mechanistic insights into how hCtr1 interacts with cDDP from initial drug acquisition at the extracellular side, through transmembrane passing, until release into cytoplasmic space, are largely unknown. Likewise, how Atox1 delivers cDDP to the secretory compartment and then exports outward from the cells by ATP7A/ATP7B efflux pumps is incompletely understood. These results underscore the need for further research to gain new insight into the roles of copper transport system and Pt-based cancer chemotherapy. Another area that has not been well explored is how the Cu transport system ultimately delivers cDDP to the nucleus, where it exerts the lethal cytotoxic consequence.

While it has been clear that Cu homeostasis plays an important role in regulating the overall cellular cDDP contents which are the critical determinant of cell killing capacities. It has been reported that hCtr1 can be transcriptionally modulated by Cu contents via the transcriptional factor Sp1. How Sp1 senses cellular Cu concentrations and regulates hCtr1 expression has been noted. However, much remains to be learned, especially how Cu homeostasis may regulate the expression and activities of Atox1 and ATPases which also regulate intracellular cDDP behavior. cDDP transport is a long and complex process in cancer pharmacology. It is anticipated that better understandings of these processes would provide valuable translational avenues for clinical applications.

Cultured cell research on hCtr1 regulation by Cu homeostasis has resulted in two clinical trials using Cu-lowering agents. While this strategy has produced encouraging results, however, it remains to be improved before broad application can be achieved. Given the considerations that the constraint of the Cu-Sp1-hCtr1 mutual regulatory loop and that both Sp1 and hCtr1 lack strong regulatable promoters for substantial transcriptional upregulation under Cu depleted conditions, this strategy may be more productive if by combining other targeting strategies such as Atox1, ATP7A and ATP7B, as a whole or in combinations. Admittedly, this remains a large uncharted area of research. In this junction, it may be important to mention the recent report of using a high throughput synthetic lethal screening of cDDP-resistant cell line that identified three FDA-approved drugs (Tranilast, Telmisartan, and Amphotericin B) that reduced cDDP resistance. All three drugs induced Pt-mediated DNA damage and inhibited trafficking of ATP7B in a tumor-specific manner [117]. It is anticipated that targeting a broader scope of the Cu transport system may eventually result in better utilizations of Pt-based drugs in cancer chemotherapy.

## Data Availability

Not applicable.

## Abbreviations

Atox1	antioxidant protein 1
ATP7A, ATP7B	two P-type ATPases involved in Cu and cDDP export
CCS	superoxide dismutase 1 Cu carrier
Ctr1	high-affinity copper transporter 1
cDDP	cisplatin (cis-diamminedichloroplatinum II)
COMMD1	the copper metabolism MURR1 domain 1
Cox17	cytochrome C oxidase copper chaperone
D-Pen.	D-penicillamine
γGCS	γ-glutamylcysteine synthesis
GSH	glutathione,
GSSG	oxidized form of GSH
Gpx	glutathione peroxidase
Gred	glutathione reductase
LRRC	leucine-rich repeat-containing protein
SOD1	superoxide dismutase 1
Sp1	specificity protein 1
OCT	organic cation transporter system
ROS	reactive oxygen species
TCG	The Cancer Genome Atlas
TGN.	Trans-golgi network
TM	tetrathiomolybdate
VRAC	volume-regulated anion channel
ZFs	zinc fingers
